# The Eight-Item Center for Epidemiological Studies Depression Scale in the English Longitudinal Study of Aging: Longitudinal and Gender Invariance, Sum Score Models, and External Associations

**DOI:** 10.1177/10731911221138930

**Published:** 2022-12-13

**Authors:** Pascal Schlechter, Tamsin J. Ford, Sharon A. S. Neufeld

**Affiliations:** 1 University of Cambridge, UK

**Keywords:** depression, older-aged populations, Center for Epidemiological Studies Depression Scale, longitudinal measurement invariance

## Abstract

The disease burden of depression among older populations is high. Detecting changes in late-life depression is predicated on the seldom-examined assumption of longitudinal measurement invariance (MI). Therefore, we investigated longitudinal MI of the 8-item Center for Epidemiological Studies Depression Scale in core members repeatedly assessed in the English Longitudinal Study of Aging, a nine-wave representative study of the English population above 50 years of age (initial *N* = 11,391). Based on prior literature, we tested MI of a one-factor solution, a one-factor solution with correlated errors of reversely coded items, and a two-factor solution (depressed affect/somatic complaints). For all factor solutions, residual MI was confirmed across nine waves and gender. Sum score models (i.e., all factor loadings constrained to equity) had a good fit. Depression scores correlated with psychiatric diagnoses, ill health, lower life quality, and female gender. Associations slightly differed depending on the factor solutions, signifying their applicability across contexts.

Depression is one of the top 10 causes of years lost due to disability in adults and is thus a major public health concern (Q. Liu et al., 2020; Vos et al., 2015). A meta-analysis revealed major depression to have an estimated prevalence of 16.5% among people aged 50 years and older in Western countries (Volkert et al., 2013). Depression in older people is commonly associated with coexisting medical illnesses and cognitive impairment (Taylor, 2014). The resulting levels of functional impairment, clinical suffering, and global disease burden necessitate an accurate assessment of symptoms in older populations (Kessler et al., 2010). To this end, the 8-item version of the Center for Epidemiological Studies Depression Scale (CES-D; Radloff, 1977) is routinely used in epidemiological research (Steptoe et al., 2013). Although the validity of the CES-D 8-item version has been demonstrated across different populations (Missinne et al., 2014), it remains unknown whether it measures the same underlying construct over time in older populations. As part of the aging process, several physiological and emotional changes take place, and symptoms that are indicative of depression in early and mid-adulthood (e.g., somatic symptoms) may be less strongly related to the underlying construct at a later point (Schaakxs et al., 2017). Establishing measurement invariance (MI) of CES-D is important to obtain unbiased estimates of depression in older populations (Djernes, 2006).

## The Center for Epidemiological Studies Depression Scale

Derived from the CES-D 20-item self-report instrument (Kim et al., 2011; Radloff, 1977; Shafer, 2006), different short forms of the CES-D have been developed and validated such as the CES-D-10 (González et al., 2017) and the CES-D-8 (O’Halloran et al., 2014). The CES-D-8 has shown similar psychometric properties to the 20-item version in representative samples of adults aged 70 and older in the United States (Turvey et al., 1999) and adults aged 50 and above in Ireland (Briggs et al., 2018). In contrast to the original four response options of the CES-D-20 (Radloff, 1977), the CES-D-8 applies a dichotomous response format to reduce participant burden and confusion resulting from the larger number of response options (Turvey et al., 1999). However, this change in response format did not affect the psychometric properties of the scale (Turvey et al., 1999). Thus, the CES-D-8 is often utilized in epidemiological studies such as the European Social Survey (Zivin et al., 2010), the Health and Retirement Study (Van de Velde et al., 2010), and the English Longitudinal Study of Aging (Steptoe et al., 2013). The reliability and validity of the CES-D-8 have been demonstrated across a range of adult populations (Karim et al., 2015; Missinne et al., 2014; Van de Velde et al., 2010).

### CES-D-8 Factor Structure

**Figure 1.**
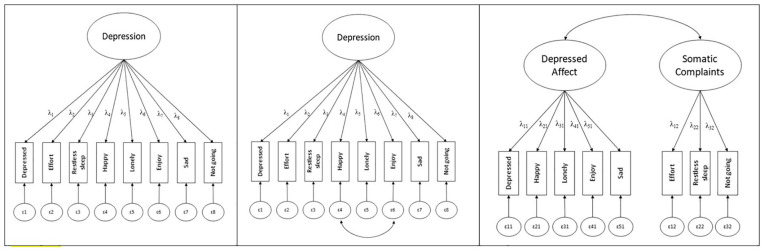
Representation of the Three Different Factor Solutions of CES-D-8 as Presented in Our Review *Note.* In the left panel, the one-factor solution is depicted (Model 1). In the middle panel, the one-factor solution is modified to allow for the correlation of reversely coded items (Model 2). The right panel depicts the two-factor solution with the two correlated factors “depressed affect” and “somatic complaints” (Model 3). CES-D = Center for Epidemiological Studies Depression Scale.

There are several different CES-D-8 factor structures reported in the literature (Figure 1). By calculating unweighted total scores, researchers often implicitly assume a one-factor model with a common latent depression factor (Levecque et al., 2011). This factor solution assumes that the shared variance among items can be captured by one underlying latent depression construct. Such a factor solution offers convenient applications for researchers because simple composite scores can be implemented in the analyses (however, note that this is based on strict assumptions which we discuss below). However, prior research shows this factor structure is not always confirmed empirically, but allowing error covariances between the two reversely coded items (*enjoyed life* and *happy*) incrementally improved model fit (Missinne et al., 2014; Van de Velde et al., 2010; for a cross-sectional study in an older population see Karim et al., 2015). The shared variance of the two reversely formulated items appears to be attributable to slightly different response patterns evoked by the different phrasing of the items (DiStefano & Motl, 2006; Lindwall et al., 2012). Using this modified single-factor solution, MI has been demonstrated cross-sectionally across 11 European countries in populations aged 50 and older (Missinne et al., 2014) and gender MI established in a sample of 25 European countries (Van de Velde et al., 2010). This demonstrates that in those aged 50 and older, CES-D-8 measures the depression construct consistently across gender and across these European countries.

Alternatively, a two-factor solution has been proposed with two distinct yet correlated factors: depressed affect *(enjoyed life, felt depressed, happy, lonely*, and *felt sad*) and somatic complaints *(everything was an effort, sleep was restless*, and *I could not get going*; e.g., Iob et al., 2020; Steffick, 2000). This factor solution allows a more nuanced distinction between symptoms and may elucidate more specific symptom profiles of depression (Shafer, 2006). For instance, depressed affect and somatic complaints may change differentially as a function of age, and levels of each will have different implications for intervention. For example, the multidimensional construct of life satisfaction may reveal unique associations with these factors: self-realization may be more negatively associated with depressed affect, while somatic complaints may be associated with reduced perceived autonomy and control (Sivertsen et al., 2015). The CES-D-8 two-factor solution fitted slightly better than the original one-factorial solution in cross-sectional research (Iob et al., 2020).

However, for each of these factor solutions, there is a lack of knowledge regarding longitudinal MI in older populations. While studies have tested temporal MI of the CES-D-20 (e.g., in mothers of children who have epilepsy; Ferro & Speechley, 2013; in middle-aged and older adults, Mogos et al., 2015), and of the 5 items of the CES-D-8 assessing depressed affect (across six waves in the English Longitudinal Study of Aging, Blöchl et al., 2022), we are not aware of any study that systematically examined MI of the full CES-D-8 scale in older adults.

## Longitudinal MI

Temporal MI testing discerns whether differences across time are attributable to differences in the latent construct or instead to differences in factor loadings, item thresholds, or error variances (Y. Liu et al., 2017). MI is established with increasingly constrained models that are consecutively tested against each other. First, the factor structure (number of factors) is constrained to be equivalent across time points (configural MI). Next, the factor loadings are constrained to be equal across time to investigate whether the items relate to the latent depression trait in the same way across time points or groups (weak/ metric MI). Then, item thresholds (for categorical data) are constrained to equity to discern whether the thresholds conditional on the latent dimension are the same across time (strong/ scalar MI). Last, the residual variances of the items are constrained to equity over time to examine residual invariance (unique factorial MI). In the context of longitudinal MI with categorical indicators, residual MI needs to be demonstrated to ensure that any changes in the means or covariances of the observed scores reflect changes in the underlying latent construct (Y. Liu et al., 2017). Longitudinal MI could be violated if the construct of depression changes over time in older people (Fiske et al., 2009). Given a higher overall prevalence of somatic symptoms in older people (Hegeman et al., 2015), these symptoms may then no longer be able to differentiate individuals on the latent construct of depression. More participants may endorse the items *everything I did was an effort, my sleep was restless*, and *I could not get going* because of physiological changes associated with aging. As a consequence, the somatic factor may change its underlying meaning across time. Alternatively, in a one-factor solution, lower factor loadings of these items could indicate they do not represent the latent construct as well, or lower thresholds could be seen, indicating that endorsing symptoms for reasons other than depression becomes more common with age. This could lead to overestimating depression prevalence as scores may be inflated when researchers count symptoms that do not reflect depression. Indeed, a review that compared available depression tools for older people concluded that well-validated scales in older populations such as the Geriatric Depression Scale (Dunn & Sacco, 1989) contain fewer somatic items than other scales (Balsamo et al., 2018). These considerations are also reflected in applied research that used the CES-D in older populations and tested the depressed affect subscale without the somatic items as a sensitivity analysis (J. White et al., 2016). In their testing of the dose–response relationship between the duration of depressive symptoms and mortality risk, these authors found no differences when excluding the somatic symptoms (J. White et al., 2016). Other studies suggest low mood is less common in older adults, indicating that reported depressive symptoms are not merely an artifact of age-related mood changes (Charles & Carstensen, 2010; Sutin et al., 2013). In this regard, the depressed affect factor may not be strongly affected by aging processes and be well-suited to differentiate between individuals on the latent trait of depression.

## Gender MI

Interpreting scores in depression scales can be complicated by possible gender differences in the endorsement of depressive symptoms. Meta-analyses show that women report higher rates of depression than men with a ratio around 2:1, a ratio that is stable from the early twenties onwards (J. S. Hyde & Mezulis, 2020; Salk et al., 2017). Such differences may be attributable to a complex combination of social, biological, and psychological factors (Anyan & Hjemdal, 2018; Lewis et al., 2018; Nolen-Hoeksema & Aldao, 2011). However, different endorsement levels could reflect different ways in which females and males report their symptoms. Although some studies have identified gender bias in longer versions of the CES-D that may artificially inflate such differences (Stommel et al., 1993), the CES-D-8 has demonstrated gender invariance in representative probability samples of those aged 15 years and older (Van de Velde et al., 2009, 2010). However, it is untested as to whether this gender invariance applies in older adults over time. This is an important gap to redress as differentially across gender certain items may be more strongly indicative of depression. For example, research indicates more profound effects of loneliness in men compared with women (Curran et al., 2020). Conversely, women may report higher degrees of loneliness because they live longer than men and may thus be more affected by widowhood. However, sole endorsement of loneliness may not reflect depression; this could lead to lower factor loadings of this symptom on the depression latent construct in older adults. Thus, at different stages of the aging process, the item “feeling lonely” may be differentially reflective of depression across gender. Likewise, there are sex differences in sleep problems, with women being affected by insomnia more often than men, attributable to complex social, psychological, and biological factors (Suh et al., 2018). Accordingly, the item “restless sleep” may not be equally reflective of depression across gender. Only if measurement properties do not deviate from each other across gender do mean-level differences reflect true score differences over time (J. S. Hyde & Mezulis, 2020; Salk et al., 2017).

## Sum Score Models

Based on reported evidence of cross-national and gender MI, sum score models have often been applied in research using the CES-D-8 (Levecque et al., 2011). However, in established MI models, symptoms relate differently to the underlying construct because they have different factor loadings (McNeish & Wolf, 2020). Deriving sum scores from such congeneric models with high variability in factor loadings of a given trait can result in biased test scores and conclusions (McNeish & Wolf, 2020). Even if longitudinal MI is established, this does not mean that sum score models can be applied. Longitudinal MI establishes that the same indicators have the same factor loadings over time. However, within a measurement wave, factor loadings vary and provide different information on the underlying construct. This information is however not contained in sum scores that treat all indicators of the underlying construct equally (McNeish & Wolf, 2020). Therefore, the CES-D-8 needs to be tested to determine whether factor models resembling the sum score (i.e., models with all factor loadings constrained to equity) can be an adequate representation of the data. Only when the model fit is still adequate under these strong assumptions, can simple unweighted sum scores be used in applied research as an approximation of the change in depression over time.

## The Present Study

We examined longitudinal and gender MI of the CES-D-8 using the English Longitudinal Study of Aging (ELSA), a nine-wave representative study of the English population aged 50 years and older (Steptoe et al., 2013). Specifically, we tested MI of depressive symptoms for the three proposed factor solutions (a one-factor solution, a one-factor solution with correlated errors of reversely coded items, and a two-factor solution) across (a) the nine waves and (b) gender (female vs. male). Furthermore, to examine the acceptability of using sum scores in applied research, we tested whether sum score models adequately represented the data. To discern the validity of these sum scores, we examined their association with external constructs. To this end, we correlated total scores (and subscale scores if relevant) with a psychiatric diagnosis, gender, general health, and life quality, all of which were associated with depression in previous studies (Salk et al., 2017; Sivertsen et al., 2015).

## Method

### Participants and Study Design

A detailed study profile of ELSA is reported in Steptoe et al. (2013). Briefly, this prospective cohort study started in 2002/2003 with follow-up surveys once every 2 years, finishing with the last wave (Wave 9) in 2018/2019. The initial cohort comprised 11,391 adults (core members) born on or before February 29, 1952. Participants were drawn from households that took part in the Health Survey for England (HSE; 1998, 1999, & 2001). Multistage stratified probability sampling was used. To maintain representativeness, the sample was refreshed in several waves with participants above 50 years from other HSE waves. For the purposes of our longitudinal MI analyses, the present study only includes core sample members who were followed throughout the nine waves of data collection (see Table 1 for the sample sizes of core sample members at each wave). Demographic characteristics of the sample consisting of core members are shown in Table 1. Ethical approval was given by the National Research Ethics Service (MREC/01/2/91). ELSA data are openly available to researchers at the U.K. Data Service.

**Table 1:** Demographic Characteristics for Each Wave.

Demographic characteristics	Wave 1(*N* = 11,391)	Wave 2 (*N* = 8,780)	Wave 3 (*N* = 7,326)	Wave 4 (*N* = 6,623)	Wave 5 (*N* = 6,242)	Wave 6 (*N* = 5,659)	Wave 7 (*N* = 4,894)	Wave 8 (*N* = 4,219)	Wave 9 *(N* = 3,660)
Age (*SD*)	65.19 (10.23)	66.81 (9.77)	68.19 (9.55)	69.65 (9.08)	70.96 (8.66)	72.25 (8.26)	73.50 (7.74)	70.74 (17.34)	72.73 (16.42)
Gender									
Female	6,205 (54%)	4,830 (55%)	4,181 (58%)	3,708 (56%)	3,500 (56%)	3,181 (56%)	2,767 (57%)	2,384 (56%)	2,092 (57%)
Male	5,186 (46%)	3,950 (45%)	3,354 (42%)	2,915 (44%)	2,742 (44%)	2,478 (44%)	2,127 (43%)	1,835 (44%)	1,568 (43)
Ethnicity									
White	11,065 (97%)	8,586 (98%)	7,382 (99%)	6,489 (98%)	6,102 (98%)	5,527 (98%)	4,784 (98%)	4,118 (98%)	3,573 (98%)
Non-White	320 (3%)	194 (2%)	153 (1%)	134 (2%)	140 (2%)	132 (2%)	110 (2%)	101 (2%)	87 (2%)
Education									
Less than secondary	4,878 (43%)	3,475 (40%)	2,856 (39%)	2,404 (36%)	2,179 (35%)	1,917 (34%)	1,544 (32%)	1,255 (30%)	1,025 (28%)
Upper secondary	4,256 (37%)	3,481 (40%)	3,055 (42%)	27,46 (41%)	2,640 (42%)	2,434 (43%)	2,205 (45%)	1,935 (46%)	1,713 (46%)
Tertiary	1,257 (20%)	1,057 (20%)	955 (19%)	902 (23%)	884 (23%)	828 (23%)	738 (23%)	677 (24%)	608 (26%)
Retirement									
Yes	5,774 (51%)	4,776 (54%)	4,488 (61%)	4,385 (73%)	4,485 (72%)	4,447 (79%)	4,043 (81%)	3,645 (86%)	3,228 (88%)
Employed									
Yes	3,607 (32%)	2,516 (29%)	1,976 (27%)	1,467 (22%)	1,107 (18%)	771 (14%)	534 (11%)	340 (8%)	233 (7%)
Marital Status									
Married	7,570 (66%)	5,882 (66%)	5,054 (69%)	4,511 (68%)	4,342 (70%)	3,987 (70%)	3,501 (72%)	3,053 (72%)	2,666 (73%)
Divorced	857 (5%)	687 (7%)	613 (8%)	535 (8%)	510 (8%)	468 (8%)	406 (8%)	364 (9%)	327 (9%)
Never married	580 (5%)	422 (4%)	361 (5%)	320 (5%)	275 (4%)	245 (4%)	205 (4%)	171 (5%)	153 (4%)
Partnered	283 (2%)	236 (3%)	198 (3%)	183 (3%)	173 (3%)	163 (3%)	154 (3%)	143 (3%)	133 (4%)
Separated	140 (1%)	103 (1%)	89 (1%)	76 (1%)	76 (1%)	70 (1%)	58 (1%)	51 (1%)	42 (1%)
Widowed	1,959 (17%)	1,448 (16%)	1,219 (17%)	977 (15%)	865 (14%)	725 (13%)	569 (12%)	436 (10%)	338 (9%)
Psychiatric diagnosis									
Yes	765 (7%)	741 (8%)	687 (9%)	661(10%)	653 (10%)	634 (11%)	576 (12%)	496 (12%)	429 (12%)

### Measures

#### Center for Epidemiologic Studies Depression Scale (CES-D)

Symptoms of depressed affect and somatic complaints in the previous week were assessed with the CES-D-8 (Turvey et al., 1999). The dichotomous (yes/no) response format results in total scores ranging between 0 (*no symptoms*) and 8 (*all eight symptoms*). Total scores of three or above suggest depression “caseness” (Turvey et al., 1999).

### External Variables

#### Psychiatric Diagnosis

A dichotomous (no/yes) self-report question assessed whether participants had been diagnosed with a psychiatric disorder during their lifetime.

#### General Health Problems

One question pertained to participants’ self-reported general health using a global assessment on a 5-point Likert-type scale from 1 (*very good*) to 5 (*very bad*; Bowling & Windsor, 2008).

#### Quality of Life

The 19-item control, autonomy, self-realization, and pleasure (CASP) measure was used (M. Hyde et al., 2003). Previous psychometric analyses of the ELSA sample concluded that the subscales control and autonomy should be combined (Wiggins et al., 2008). We, therefore, used this combined scale and the self-realization and pleasure subscales alongside a sum score of all items. Internal consistencies (Cronbach’s α) of these four derived scores in our sample ranged from .79 to .91.

### Missingness

Analyses included only core sample members that participated in Wave 1 (*N* = 11,391). There was substantial attrition during the study with *N* = 3660 core members participating in wave nine, resulting in missingness rates up to 69% (Table 1). Little’s test for missing data (Little, 1988) indicated that data were not missing completely at random, *p* < .001. Missingness was predicted by being non-White, older, unmarried, having a lower level of formal education, and having more depressive symptoms at Wave 1, all *p* < .001 (previously reported in Lee et al., 2021 and J. White et al., 2016). Accordingly, we concluded that data were missing depending on observed variables (Missing at Random; MAR). The weighted least squares mean and variance adjusted (WLSMV) estimator required for the present dichotomous data is a limited-information estimator (Asparouhov & Muthén, 2010). Therefore, to ensure unbiased parameter estimates under these assumptions (Graham, 2009), we conducted multiple imputation by chained equations using the MICE package in *R* (van Buuren et al., 2015). We used logistic regression to impute the values of the dichotomous indicators (I. R. White et al., 2011). Five imputed data sets were produced using demographic variables that were associated with missingness as auxiliary variables in our imputation model. This number of imputations was chosen as it enables precise point estimates while reducing computational demands (I. R. White et al., 2011), and accurate standard errors were not required for the present MI analyses. Sensitivity analyses were conducted on complete cases (core sample members) that provided data throughout the nine waves (without auxiliary variables).

## Analysis Strategy

Analyses were performed in R Version 3.14 (*R* Core Team, 2019). MI analyses were conducted with the lavaan package in *R* (Rosseel, 2012). Imputations were performed in lavaan using the function cfa.mi, which pools the results directly with imputed data sets generated with the MICE package. Given the dichotomous nature of our response options, we used WLSMV for all models. Weighted least squares approaches (Muthén, 1984) use item thresholds to account for the ordered nature of the observed data. In these approaches, it is assumed that participants’ responses reflect a discrete categorization of the underlying latent variable and that both are related by a threshold relationship. An observed variable with *r* response categories has *r*-1 thresholds (τ_j_), resulting in one threshold for the dichotomous response options of the CES-D. Parameter estimates are then based on thresholds and tetrachoric correlations among the dichotomous items using a least square fit function. In the WLSMV approach, the mean and variance of the chi-square test statistic are adjusted to approximate the expected distribution more accurately than unadjusted approaches. According to simulation studies, WLSMV produce sufficiently accurate model parameter estimates with dichotomous response options (Liang & Yang, 2014; Moshagen & Musch, 2014).

### Factor Models

#### Model 1: One-Factor Solution

Based on support for a one-factor solution (Levecque et al., 2011), we first tested the MI of this model across all nine waves (see Figure 1).

#### Model 2: One-Factor Solution With Correlated Errors

Second, we investigated MI for the same model but allowed the covariance of the measurement errors between the 2 items that were initially reversely coded (*happy* and *enjoying life*), as recommended by Van de Velde et al. (2010).

#### Model 3: Two-Factor Solution

Third, we tested MI for the two-factor solution with the depressed affect and the somatic complaints factors (Steffick, 2000).

#### Additional Model Constraints

Fourth, we put additional multigroup constraints for gender on models 1-3 with the highest established level of longitudinal MI. Fifth, we tested how well the one- and two-factor solutions resembled sum score models, by constraining the factor loadings of each factor to be equal and testing this against the unconstrained model.

### MI Testing

To establish MI, increasingly constrained models were tested against each other (Y. Liu et al., 2017). First, the factorial structure was constrained to be invariant across time to indicate configural invariance. Second, as recommended for categorical data (B. Muthén & Asparouhov, 2002), we next tested scalar invariance by constraining the factor loadings and item thresholds of the same indicators to be equal across waves. Constraining factor loadings and thresholds at the same time is based on the premise that the probability of endorsing an item response category is jointly determined by the factor loadings and thresholds (for more details see Chen et al., 2020; L. K. Muthén & Muthén, 2017; Sass et al., 2014; Stark et al., 2006). Third, item error variances were constrained to equity over time to examine residual MI. In all models, autocorrelated residuals were allowed. The following criteria were applied: a Comparative Fit Index (CFI) above .95 and Root Mean Square Error of Approximation (RMSEA) below .05 indicate good model fit (Hu & Bentler, 1998). Adequate pooling procedures for these fit indices across multiple imputed data sets have not yet been established (Y. Liu et al., 2017, 2021; Y. Liu & Sriutaisuk, 2021; Shi et al., 2020). We therefore used naive averages of these fit indices across imputations to evaluate model fit. Simulation studies with ordered factor models suggest that the CFI seems to be relatively unbiased when using the naïve average across imputations, while the RMSEA may be slightly overestimated when levels of missingness are high (Shi et al., 2020). Changes in these indices indicated MI: the ΔCFI should be <.010 and the ΔRMSEA <.007 when tested against the model established in the prior step (Neufeld et al., 2022).^1^ We did not investigate χ^2^ differences because they are likely significant given our large sample size (Y. Liu et al., 2017; Neufeld et al., 2022). To ensure model identification for all models, we have followed the steps outlined by Edossa et al. (2018) and applied theta instead of delta parameterization. If MI was not established, we tested partial MI by relaxing constraints on parameters that deviated strongly according to modification indices above a cutoff of 10 (Byrne et al., 1989; Guenole & Brown, 2014).

### External Validation

To elucidate time-varying associations of the different factor solutions, we correlated the CES-D-8 total and subscale scores with gender, age and concurrent psychiatric diagnosis, general health status, and quality of life, which were all assessed at the same waves. Because we aimed to investigate the differential effects of the two factors (depressed affect and somatic complaints), we calculated 95% confidence intervals around the point estimates (Cumming & Finch, 2005; Greenland et al., 2016). To obtain accurate standard errors for these analyses, we imputed 70 data sets to account for the rate of missingness (up to 69%; I. R. White et al., 2011). Again, we included the demographic variables that were associated with missingness as auxiliary variables.

## Results

### Descriptive Statistics

Table 2 displays the percentage of participants who endorsed each item per wave. The level of endorsement was consistent across waves. The symptom *restless sleep* consistently had the highest endorsement. *Being happy* and *enjoying life* had the lowest endorsement following recoding. Internal consistencies were excellent, and Cronbach’s α (α) and Omega total (*ω_t_*) were consistently ≥.90. Most items deviated from normality, displaying positive skew above 1 (after recoding as appropriate) and kurtosis above 3.

**Table 2.** Percentage of Participants Indicating Yes on the Dichotomous CES-D Items, Sum Scores for Each Wave and Internal Consistencies.

During the past week indicate whether you. . .	Wave 1 (*N* = 11,391)	Wave 2 (*N* = 8,780)	Wave 3 (*N* = 7,326)	Wave 4 (*N* = 6,623)	Wave 5 (*N* = 6,242)	Wave 6 (*N* = 5,659)	Wave 7 (*N* = 4,894)	Wave 8 (*N* = 4,219)	Wave 9 (*N* = 3,660)
1 . . .you felt depressed?	17.92	16.47	15.06	14.49	14.42	12.38	11.76	12.24	11.65
2 . . .you felt everything you did was an effort?	23.97	22.70	21.23	19.55	21.36	19.27	20.17	20.73	20.61
3. . . .your sleep was restless?	40.97	42.35	40.73	33.74	40.21	32.85	39.80	35.16	42.48
4. . . .you were happy?	88.93	89.52	89.81	90.07	89.96	90.65	90.92	91.68	91.35
5. . . .you felt lonely?	13.83	14.15	13.83	13.49	14.26	12.32	11.67	12.26	11.86
6. . . .you enjoyed life?	90.26	90.14	90.60	90.72	90.15	90.73	91.49	92.46	91.47
7. . . .you felt sad?	20.74	21.46	19.22	20.05	20.93	17.87	16.63	19.42	18.42
8. . . .you could not get going?	22.01	21.35	21.63	20.31	22.32	19.43	20.84	20.85	20.73
Sum score (*SD*)	1.61 (2.00)	1.59 (1.96)	1.52 (1.96)	1.41 (1.91)	1.54 (1.96)	1.33 (1.85)	1.39 (1.83)	1.36 (1.78)	1.42 (1.78)
Cronbach’s alpha (α)	.92	.91	.92	.92	.92	.92	.91	.90	.90
Omega total (ω_t_)	.93	.92	.93	.92	.92	.92	.92	.90	.91
Skewness/kurtosis	Wave 1 (*N* = 11,391)	Wave 2 (*N* = 8,780)	Wave 3 (*N* = 7,326)	Wave 4 (*N* = 6,623)	Wave 5 (*N* = 6,242)	Wave 6 (*N* = 5,659)	Wave 7 (*N* = 4,894)	Wave 8 (*N* = 4,219)	Wave 9 *(N* = 3,660)
1 . . .you felt depressed?	1.67/ 0.80	1.81/ 1.27	1.95/ 1.82	2.02/ 2.02	2.05/ 2.18	2.28/ 3.22	2.37/ 3.63	2.30/ 3.31	2.39/ 3.71
2 . . .you felt everything you did was an effort?	1.22/−0.51	1.30/−0.30	1.41/−0.02	1.54/ 0.36	1.40/−0.05	1.56/ 0.43	1.49/ 0.21	1.44/ 0.88	1.45/ 0.11
3. . . .your sleep was restless?	0.37/−1.87	0.31/−1.90	0.38/−1.86	0.69/−1.53	0.40/−1.84	0.73/−1.47	0.42/−1.83	0.62/−1.61	0.30/−1.91
4. . . .you were happy?	−2.48/ 4.16	−2.58/ 4.66	−2.63/ 4.92	−2.68/ 5.18	−2.66/ 5.07	−2.79/ 5.08	−2.85/ 6.11	−3.02/ 7.10	−2.94/ 6.65
5. . . .you felt lonely?	2.09/ 2.39	2.06/ 2.23	2.10/ 2.39	2.14/ 2.57	2.04/ 2.18	2.29/ 3.26	2.39/ 3.70	2.30/ 3.29	2.36/ 3.57
6. . . .you enjoyed life?	−2.72/ 5.37	−2.69/5.25	−2.78/ 5.74	−2.81/5.88	−2.69/ 5.25	−2.81/ 5.88	−2.97/ 6.83	−3.21/ 8.33	−2.97/ 6.81
7. . . .you felt sad?	1.44/ 0.08	1.39/−0.07	1.56/ 0.44	1.50/ 0.24	1.43/ 0.04	1.68/ 0.81	1.79/ 1.21	1.55/ 0.39	1.63/ 0.65
8. . . .you could not get going?	1.35/−0.18	1.40/−0.05	1.38/−0.10	1.48/ 0.18	1.33/−0.23	1.54/ 0.39	1.44/ 0.06	1.43/ 0.06	1.44/ 0.08

*Note.* CES-D = Center for Epidemiological Studies Depression Scale.

### Factor Models

Table 3 displays freely estimated factor loadings and thresholds for both the one-factorial and two-factorial solutions. Factor loadings were consistent in their estimates across the nine waves. *Restless sleep* showed the most deviation, peaking at .15 and .12 for the one- and two-factorial solution, respectively. This symptom also displayed the lowest factor loading across waves, but the magnitude of loadings was still acceptable. The remaining items deviated by no more than .08 for factor loadings over time. For the one-factorial solution, *feeling depressed* consistently had the highest loadings, followed by *everything was an effort.* These two symptoms also showed the highest loadings on their respective factors in the two-factorial solution.

**Table 3.** Freely Estimated Factor Loadings and Item Thresholds (Modified One-Factorial/Two-Factorial) Solution.

Freely estimated factor loadings	λ_k1_	λ_k2_	λ_k3_	λ_k4_	λ_k5_	λ_k6_	λ_k7_	λ_k8_	λ_k9_	*D*_max_
1 . . .you felt depressed?	.86/ .91	.87/ .91	.88/ .92	.84/.89	.81/.86	.86/ .91	.87/.92	.85/.88	.88/ .90	.07/.06
2 . . .**you felt everything you did was an effort?**	.85/ .88	.85/ .90	.85/ .91	.83/ .88	.87/ .92	.85/ .90	.82/ .89	.79/ .88	.83/ .91	.08/.04
3. . . .**your sleep was restless?**	.61/ .61	.54/.56	.54/ .55	.59/ .61	.52/ .53	.50/ .51	.49/ .52	.49/ .53	.46/ .49	.15/.12
4. . . .you were happy?	−.80/−.84	−.77/−.80	−.78/−.81	−.77/−.81	−.76/−.80	−.76/−.79	−.76/−.80	−.74/−.76	−.81/ −.83	.05/.08
5. . . .you felt lonely?	.72/ .76	.75/ .79	.77/ .80	.76/ .80	.77/ .82	.72/ .76	.74/ .79	.72/ .74	.77/ .79	.05/.08
6. . . .you enjoyed life?	−.81/−.85	−.84/−.88	−.84/−.87	−.83/−.87	−.81/−.85	−.82/−.86	−.77/−.81	−.77/ −.79	−.80/−.82	.07/.08
7. . . .you felt sad?	.77/ .82	.71/ .75	.76/ .80	.72/ .76	.74/. 78	.72/ .75	.75/ .79	.74/ .76	.76/ .78	.06/.07
8. **. . .you could not get going?**	.82/ .84	.76/ .79	.78/ .82	.80/ .84	.78/ .82	.80/ .84	.77/ .83	.76/ .83	.75/ .81	.07/.05
Freely estimated thresholds	τ_k1_	τ_k2_	τ_k3_	τ_k4_	τ_k5_	τ_k6_	τ_k7_	τ_k8_	τ_k9_	*D*_max_
1 . . .you felt depressed?	0.92	0.98	1.04	1.05	1.07	1.12	1.14	1.06	1.01	0.22
2 . . .you felt everything you did was an effort?	0.71	0.74	0.79	0.83	0.75	0.78	0.74	0.64	0.59	0.24
3. . . .your sleep was restless?	0.23	0.20	0.23	0.42	0.23	0.46	0.23	0.35	0.13	0.33
4. . . .you were happy?	−1.22	−1.25	−1.28	−1.26	−1.26	−1.28	−1.25	−1.26	−1.13	0.13
5. . . .you felt lonely?	1.09	1.07	1.09	1.08	1.06	1.09	1.10	1.04	0.98	0.11
6. . . .you enjoyed life?	−1.29	−1.28	−1.32	−1.31	−1.28	−1.24	−1.27	−1.31	−1.13	0.18
7. . . .you felt sad?	0.82	0.79	0.88	0.83	0.82	0.86	0.91	0.81	0.77	0.14
8. . . .you could not get going?	0.77	0.78	0.77	0.80	0.71	0.81	0.71	0.68	0.63	0.18

*Note. K* refers to the number of the item. Bolded items constitute the somatic factor. *D*_max_ = maximal deviation of factor loading/ thresholds across waves.

Freely estimated thresholds were consistent across waves without strong deviations (see Table 3) Again, the strongest deviation was found for *restless sleep.* This symptom also displayed the lowest threshold, suggesting that respondents endorsed this relatively easily compared to other symptoms. Both reversely coded items also displayed low thresholds. *Feeling depressed* displayed the highest threshold that needed to be surpassed for the *yes* response option.

### MI Testing

Across the five imputations, values for the CFI and RMSEA were very similar with a maximum deviation of .01 for the same model.

#### Model 1: One-Factor Solution

According to CFI and RMSEA, all single-factor models displayed excellent model fit (Table 4). No deterioration in overall fit was detected according to ∆CFI and ∆RMSEA when models were increasingly constrained. We thus established residual longitudinal MI. Residual MI was also established across gender.

**Table 4.** Measurement Invariance Models.

	Full sample					Gender				
Factor model	χ^2^(df)	CFI	RMSEA	∆CFI	∆RMSEA	χ^2^(df)	CFI	RMSEA	∆CFI	∆RMSEA
1 Factor (Model 1)										
Configural	28,860.32 (2,160)	.982	.033			30,227.98 (4,320)	.982	.032		
Scalar	29,498.96 (2,208)	.981	.031	.001	.002	31,364.94 (4,431)	.981	.033	.001	.001
Residual	31,403.68 (2272)	.980	.033	.001	.002	34,362.89 (4,559)	.979	.034	.002	.001
Sum score	62,152.14 (2,230)	.959	.049	.023	.016					
Modified 1 Factor^a^ (Model 2)										
Configural	23,393.55 (2,151)	.985	.029			24,836.16 (4,302)	.985	.029		
Scalar	24,037.39 (2,199)	.985	.030	.000	.001	26,267.67 (4,413)	.985	.029	.000	.000
Residual	31,403.68 (2,272)	.980	.034	.005	.004	27,927.47 (4,478)	.983	.030	.002	.001
2 Factors (Model 3)										
Configural	14,330.02 (2,043)	.992	.023			15,373.83 (4,086)	.992	.022		
Scalar	14,552.47 (2,075)	.991	.023	.001	.000	16,148.27 (4,164)	.992	.022	.000	.000
Residual	15,834.70 (2,139)	.991	.024	.000	.001	23,306.73 (4,292)	.987	.028	.005	.004
Sum Score	56,425.34 (2,214)	.963	.046	.029	.023					

*Note*. ΔCFI ≥ .010 and ΔRMSEA ≥ .007 indicate substantial deterioration in model fit (Neufeld et al., 2022). Models are compared with the prior model consisting of one less level of constraints. CFI = Comparative Fit Index; RMSEA = Root Mean Square Error of Approximation; *df* = degrees of freedom.

a Here, we allowed for the covariances of errors between the reversely coded items.

#### Model 2: One-Factor Solution With Correlated Errors

Likewise, residual invariance was established when error covariances between the 2 reversely coded items were modeled. This error covariance model had very slight improvements in fit as compared to the initial one-factorial solution. We also found evidence for residual MI across gender.

#### Model 3: Two-Factor Solution

The two-factor solution yielded an excellent fit for all models with a better fit than both one-factorial solutions. Residual longitudinal and gender MI was established. Sensitivity analyses of raw data using complete cases that provided data for all nine waves did not lead to different conclusions compared with the imputed data (Supplemental Table S1).

#### Sum Score Models

The models resembling sum scores for the one- and two-factorial solutions had worse model fit than the congeneric unconstrained model. However, the overall model fit was still good according to CFI and RSMEA.

#### Associations Over Time

Total and subscale scores from the sum score models across all waves were significantly autocorrelated (*r* = .31–.58, Table S2). Total scores were also strongly concurrently related to the respective scores of the two subdimensions depressive affect (*r* = .88–.91) and somatic symptoms (*r* = .83–.85). Concurrent correlations between depressive affect and somatic symptoms subscales revealed related but distinct constructs (*r* = .47–.53).

### External Associations

The derived sum scores of the one-factorial solution and the two-factorial solution were positively associated with lifetime psychiatric diagnosis and age across all waves expect Waves 8 and 9 (Table 5). Females had higher scores for all factors across all waves. At the first five waves, the diagnosis was more strongly associated with the affective factor than with the somatic factor (for confidence intervals see Table 5). Self-rated health problems were associated with all scores but had higher associations with the somatic factor than with the depressed affect factor. All quality-of-life subscales displayed significant associations. The overall quality-of-life score (at the first six waves) and self-realization subscales (all waves) showed stronger associations with the affective factor. The lack of pleasure (all but Waves 2 and 4) and lack of control/autonomy scales (Waves 1 and 3) were more strongly associated with somatic symptoms.

**Table 5:** Estimates and 95% Confidence Intervals (CIs) for the Associations Between Sum Scores (Total and Two-Factors) and External Constructs.

	Diagnosis			Age			Gender (m)		
Wave	1 Factor	Affective	Somatic	1 Factor	Affective	Somatic	1 Factor	Affective	Somatic
Wave 1	.18 [.16, .20]	**.17 [.16**, **.19]**	**.14 [.12, .16]**	.08 [.06, .10]	.06 [.04, .08]	.07 [.06, .09]	−.46 [−.54, −.39]	−.26 [−.31, −.21]	−.21 [−.25, −.17]
Wave 2	.17 [.15, .29]	**.18 [.15, .20]**	**.10 [.08, .12]**	.07 [.05, .09]	.06 [.04, .09]	.06 [.04, .08]	−.39 [−.46, −.31]	−.22 [−.27, −.17]	−.17 [−.21, −.13]
Wave 3	.18 [.15, .21]	**.17 [.14, .20]**	**.12 [.09, .15]**	.08 [.05, .10]	.06 [.04, .09]	.06 [.04, .08]	−.35 [−.43, −.28]	−.19 [−.24, −.14]	−.17 [−.20, −.12]
Wave 4	.16 [.13, .19]	**.15 [.12, .19]**	**.10 [.07, .12]**	.08 [.05, .10]	.06 [.03, .10]	.06 [.03, .09]	−.33 [−.40, −.25]	−.19 [−.24, −.13]	−.14 [−.19, −.10]
Wave 5	.14 [.10, .18]	**.14 [.10, .19]**	**.08 [.05, .11]**	.08 [.05, .11]	.06 [.02, .09]	.07 [.04, .10]	−.24 [−.32, −.16]	−.12 [−.18, −.07]	−.12 [−.16, −.07]
Wave 6	.14 [.11, .17]	.11 [.08, .14]	.10 [.08, .13]	.08 [.05, .10]	.06 [.03, .09]	.06 [.03, .09]	−.26 [−.33, −.18]	−.13 [−.18, −.08]	−.13 [−.17, −.08]
Wave 7	.14 [.11, .17]	.12 [.09, .15]	.09 [.07, .12]	.09 [.07, .11]	.06 [.03, .09]	.08 [.05, .11]	−.22 [−.30, −.15]	−.10 [−.15, −.05]	−.12 [−.17, −.07]
Wave 8	.03 [−.19, .15]	.02 [−.15, .19]	.06 [−.17, .29]	.01 [−.02, .04]	.02 [−.02, .05]	.00 [−.03, .04]	−.20 [−.27, −.13]	−.09 [−.15, −.04]	−.10 [−.15, −.06]
Wave 9	.02 [−.10, .13]	.00 [−.10, .09]	.03 [−.13, .19]	.02 [−.02, .06]	.01 [−.04, .06]	.02 [−.01, .06]	−.20 [−.27, −.12]	−.11 [−.16, −.06]	−.09 [−.13, −.04]
	Ill-health			Lack of control/autonomy			Self-realization		
	1 Factor	Affective	Somatic	1 Factor	Affective	Somatic	1 Factor	Affective	Somatic
Wave 1	.38 [.37, .40]	**.26 [.25, .29]**	**.42 [.39, .43]**	−.28 [−.30, −.26]	**–.18 [−.21, −.16]**	**−.24 [−.27, −.23]**	.36 [.34, .38]	**.34 [.32, .36]**	**.27 [.25, .29]**
Wave 2	.37 [.35, .39]	**.24 [.22, .26]**	**.39 [.37, .41]**	−.17 [−.20, −.15]	−.14 [−.16, −.11]	−.15 [−.18, −.13]	.32 [.29, .34]	**.31 [.29, .34]**	**.20 [.18, .22]**
Wave 3	.36 [.31, .40]	**.23 [.20, .26]**	**.38 [.31, .44]**	−.14 [−.17, −.11]	**−.10 [−.12, −.07]**	**−.14 [−.16, −.11]**	.28 [.26, .31]	**.27 [.24, .30]**	**.19 [.16, .21]**
Wave 4	.35 [.32, .38]	**.20 [.18, .23]**	**.39 [.35, .43]**	−.10 [−.13, −.08]	−.08 [−.10, −.04]	−.10 [−.07, −.12]	.24 [.21, .27]	**.22 [.19, .25]**	**.16 [.13, .18]**
Wave 5	.31 [.24, .39]	**.17 [.15, .20]**	**.35 [.23, .47]**	−.10 [−.13, −.07]	−.07 [−.10, −.04]	−.10 [−.12, −.07]	.27 [.24, .30]	**.26 [.23, .29]**	**.17 [.14, .19]**
Wave 6	.31 [.25, .36]	**.16 [.13, .19]**	**.34 [.28, .41]**	−.09 [−.12, −.06]	−.06 [−.09, −.03]	−.09 [−.11, −.06]	.25 [.22, .29]	**.24 [.20, .27]**	**.16 [.13, .18]**
Wave 7	.32 [.30, .35]	**.15 [.12, .18]**	**.38 [.35, .40]**	−.07 [−.11, −.04]	−.04 [−.08, −.02]	−.07 [−.10, −.04]	.23 [.19, .26]	**.22 [.18, .26]**	**.13 [.09, .16]**
Wave 8	.31 [.26, .35]	**.12 [.08, .15]**	**.37 [.31, .44]**	−.07 [−.09, −.04]	−.04 [−.07, −.01]	−.06 [−.09, −.03]	.19 [.15, .22]	**.18 [.14, .22]**	**.10 [.07, .13]**
Wave 9	.27 [.25, .30]	**.11 [.07, .14]**	**.32 [.29, .36]**	−.07 [−.10, −.04]	−.05 [−.08, −.02]	−.06 [−.09, −.03]	.21 [.17, .25]	**.20 [.16, .24]**	**.11 [.07, .14]**
	Loss of pleasure			Quality of life					
	1 Factor	Affective	Somatic	1 Factor	Affective	Somatic			
Wave 1	.44 [.42, .46]	**.37 [.35, .39]**	**.41 [.39, .43]**	.23 [.21, .25]	**.23 [.21, .25]**	**.17 [.15, .19]**			
Wave 2	.37 [.35, .40]	.30 [.28, .33]	.32 [.30, .34]	.20 [.18, .23]	**.19 [.17, .22]**	**.15 [.12, .17]**			
Wave 3	.32 [.29, .34]	**.24 [.22, .27]**	**.28 [.26, .31]**	.19 [.17, .21]	**.17 [.15, .20]**	**.13 [.11, .15]**			
Wave 4	.27 [.24, .30]	.21 [.18, .23]	.23 [.20, .26]	.17 [.14, .19]	**.15 [.13, .17]**	**.12 [.09, .14]**			
Wave 5	.28 [.25, .32]	**.21 [.18, .24]**	**.25 [.22, .29]**	.19 [.16, .21]	**.17 [.14, .19]**	**.13 [.11, .15]**			
Wave 6	.28 [.25, .31]	**.20 [.17, .23]**	**.24 [.21, .27]**	.18 [.15, .20]	**.16 [.13, .18]**	**.12 [.10, .15]**			
Wave 7	.24 [.20, .28]	**.16 [.12, .20]**	**.22 [.19, .25]**	.15 [.11, .18]	.12 [.09, .16]	.10 [.07, .13]			
Wave 8	.22 [.18, .26]	**.15 [.11, .18]**	**.20 [17, .24]**	.13 [.10, .16]	.11 [.08, .14]	.09 [.06, .12]			
Wave 9	.17 [.13, .21]	**.09 [.06, .13]**	**.17 [.12, .21]**	.11 [.08, .14]	.09 [.06, .11]	.08 [.05, .11]			

*Note.* Diagnosis and gender estimates display polyserial correlation coefficients. Age estimates display Pearson’s correlation coefficients (all standard errors ≤ 0.06). Females are coded with 0, males are coded with 1. Point estimates and confidence intervals are based on 70 imputations. Bolded correlations indicate *p* < .05 when comparing affective versus somatic correlations. Significant difference determined as follows: (a) *p* < .01 if non-overlapping CIs, and the margins of error do not differ by more than a factor of 2 (Cumming & Finch, 2005); (b) *p* > .05 if overlapping CIs and one of the CIs contains the point estimate from the other group (Greenland et al., 2016); (c) *p* < .05 when the overlap of the 95% CIs is no more than half the average margin of error, and the margins of error do not differ by more than a factor of 2 (Cumming & Finch, 2005).

## Discussion

In a representative cohort study, we performed longitudinal MI analyses over a time span of 16 years to understand whether CES-D-8 scores in older people represent the same latent construct across time. For all three-factor solutions (one-factor, one-factor with correlated errors of reversely coded items, and two factors), residual longitudinal MI could be established. This adds evidence to former studies that supported each of these solutions in different populations (Iob et al., 2020; Steffick, 2000; Van de Velde et al., 2010). The model fit of the one-factor solution slightly improved when we allowed the error covariance of reversely coded items, consistent with former research (Missinne et al., 2014; Van de Velde et al., 2010). These reversely coded symptoms may evoke slightly different response tendencies (DiStefano & Motl, 2006; Lindwall et al., 2012).

Descriptively, the two-factor solution showed the best fit model fit across time, which allows researchers to conduct fine-grained analyses by scrutinizing distinct symptom profiles (Fried & Nesse, 2015). This is consistent with many other depression scales that include a somatic facet (Shafer, 2006) and the original 20-item version of the CES-D that explicitly incorporated such a factor (Radloff, 1977). Our external validation scales pointed to the potential of using the two factors to disentangle differential associations. For example, the somatic factor was more strongly associated with worse self-rated health but the depressed affect factor with a psychiatric diagnosis, clearly demarcating these factors by physical and mental health problems. When considering life quality, the somatic factor was more strongly associated with less control/autonomy, which could be related to perceived lifestyle restrictions associated with somatic symptoms. On the contrary, overall quality of life and self-realization were more strongly negatively associated with the depressed affect factor, pointing to the importance of affective symptoms for life quality (for a review see Sivertsen et al., 2015).

Establishing longitudinal MI alone does not provide unequivocal justification for the use of sum scores. Testing sum score models independently is important given that depression is a heterogeneous disorder and symptoms are not always interchangeable indicators of depression (Fried & Nesse, 2015). Models based on assumptions of sum scores fit less well than the congeneric unconstrained models. However, as all these models which do not contain information on different factor loadings still had a good fit, the more easily derived sum scores can be used by researchers instead of factor scores. Our study adds justification to the use of sum scores for both tested factorial solutions. Researchers have used both in recent analyses with the ELSA data using sum scores of the two subscales (Iob et al., 2020) and applying single sum scores across time (Lee et al., 2021). Nonetheless, it should be noted that more complex factor models are the most accurate representation of the data.

Residual temporal MI of all factorial solutions provides evidence for the interpretability of change in depression over time as “true” changes in the latent construct (Y. Liu et al., 2017). Importantly, the somatic symptoms did not change in their factor loadings or thresholds over time. This is relevant because coexisting medical conditions in older people make it often more complicated to distinguish between somatic symptoms attributable to depression or other causes (Schaakxs et al., 2017). In addition, we found evidence for residual MI across gender when adding gender constraints onto the residual longitudinal MI constraints. This adds longitudinal evidence to cross-national findings that the CES-D-8 measures depressive symptoms without gender bias (Van de Velde et al., 2009, 2010). Across waves, *restless sleep* had consistently lower factor loadings than other items. This is in contrast to a cross-sectional study of CES-D-8 in a younger sample (general population aged 15 and older) where *restless sleep* was equivalent to other items in indicating the latent construct (Van de Velde et al., 2009). In the present study, *restless sleep* also had the highest endorsement and lowest threshold, consistent with a previous cross-sectional CES-D-8 study among older adults approximately 70 years of age (Karim et al., 2015). Accordingly, this item may not be ideally suited to reveal interindividual differences in depression in older populations. This could be attributable to an overall higher prevalence and complexity of sleep problems in older populations (Ancoli-Israel, 2009). This aligns with findings that symptoms associated with physical conditions like loss of appetite, loss of pleasure, cognitive decline, and sleep disturbances are easily confused with depressive symptoms in older people (Balsamo et al., 2018). Counting *restless sleep* in a cumulative sum score approach may thus inflate total scores. This problem may, however, be negligible because the overall model fit was not affected, and the factor loadings of *restless sleep* were still acceptable. Moreover, attributing somatic symptoms to medical conditions when depressive symptoms are actually present may lead to consequential underdiagnosis of depression (Barry et al., 2012). Also, this symptom is of therapeutic relevance because sleep disturbance is associated with depression treatment outcomes (Troxel et al., 2011).

Our sensitivity analyses with complete cases confirmed our conclusions regarding MI, which increases confidence in our findings. This is in line with simulation studies indicating that for MI testing, the WLSMV estimator (without auxiliary variables) produces relatively unbiased parameters and standard error estimates with up to 50% MAR missingness when the sample size is ≥1,000 (Chen et al., 2020). Model fit was descriptively slightly better for complete cases yet still very good for all models when imputed data were used. This is important because adequate pooling procedures for these fit indices across multiple imputed data sets have not yet been established (Y. Liu et al., 2017, 2021; Y. Liu & Sriutaisuk, 2021; Shi et al., 2020).

Attrition was large and of concern. While we identified variables that were associated with missingness, unmeasured variables may have influenced attrition (Graham, 2009). For instance, those with more depressive symptoms dropped out over the course of the study, and we suspect that this may be exacerbated by psychiatric comorbidity (Pierce et al., 2021). Also, a systematic review across longitudinal studies concluded that different forms of cognitive impairment predict drop-out (Chatfield et al., 2005). Furthermore, socioeconomic deprivation may have influenced attrition (e.g., Pierce et al., 2021). Parameter estimates of our imputed data are only unbiased under the assumption of MAR. There is also no clear consensus on how to establish MI across time with categorical data (see Y. Liu et al., 2017; Neufeld et al., 2022). Using the c2—test statistics or difference tests may lead to inflated Type 1 error rates, especially with large sample sizes. Changes in fit indices have not been conclusively examined (Sass et al., 2014). This may not be the largest concern in our study because all models revealed excellent fit. By demonstrating residual MI, we provide strong evidence for the internal validity of the CES-D. However, we could not include a broad range of external measures tapping into mental health outcomes to more strongly establish external validity as has previously been done with the CES-D total score (Briggs et al., 2018).

## Conclusion

With the use of representative data in older people in the United Kingdom, our study adds evidence to the excellent psychometric functioning of the CES-D-8. For all factor solutions, meaningful comparisons in depression scores across time and gender seem justified. This is critical, given the levels of functional impairment and clinical suffering associated with depression in older populations.

## Supplemental Material

sj-docx-1-asm-10.1177_10731911221138930 – Supplemental material for The Eight-Item Center for Epidemiological Studies Depression Scale in the English Longitudinal Study of Aging: Longitudinal and Gender Invariance, Sum Score Models, and External Associations Click here for additional data file. Supplemental material, sj-docx-1-asm-10.1177_10731911221138930 for The Eight-Item Center for Epidemiological Studies Depression Scale in the English Longitudinal Study of Aging: Longitudinal and Gender Invariance, Sum Score Models, and External Associations by Pascal Schlechter, Tamsin J. Ford and Sharon A. S. Neufeld in Assessment

## Notes

1. There is currently no consensus regarding these criteria. These cutoffs have been deemed suitable for categorical indicators in a review of longitudinal MI (Neufeld et al., 2022).
