# Supplementary material for: The Eight-Item Center for Epidemiological Studies Depression Scale in the English Longitudinal Study of Aging: Longitudinal and Gender Invariance, Sum Score Models, and External Associations
Source: Assessment. 2022 Dec 13;30(7):2146–61. doi: 10.1177/10731911221138930 (PMC10476547; doi:10.1177/10731911221138930)
Supplement: sj-docx-1-asm-10.1177_10731911221138930 – Supplemental material for The Eight-Item Center for Epidemiological Studies Depression Scale in the English Longitudinal Study of Aging: Longitudinal and Gender Invariance, Sum Score Models, and External Associations [file sj-docx-1-asm-10.1177_10731911221138930.docx]

|  | Full Sample | | | | | Gender | | | | |
| --- | --- | --- | --- | --- | --- | --- | --- | --- | --- | --- |
|  | $\chi^{2}(df)$ | CFI | RMSEA | ∆CFI | ∆RMSEA | $\chi^{2}(df)$ | CFI | RMSEA | ∆CFI | ∆RMSEA |
| **1 Factor (Model1)** |  |  |  |  |  |  |  |  |  |  |
| Configural | 5497.28 (2160) | .990 | .024 |  |  | 7021.39 (4320) | .991 | .022 |  |  |
| Scalar | 5641.56 (2208) | .990 | .024 | .000 | .000 | 7512.85 (4431) | .990 | .023 | .001 | .001 |
| Residual | 6017.99 (2272) | .989 | .025 | .001 | .001 | 8269.67 (4559) | .988 | .025 | .002 | .002 |
| **Modified 1 Factor* (Model 2)** |  |  |  |  |  |  |  |  |  |  |
| Configural | 4628.98 (2151) | .993 | .021 |  |  | 6184.07 (4302) | .994 | .018 |  |  |
| Scalar | 4789.05 (2199) | .992 | .021 | .001 | .000 | 6696.36 (4413) | .993 | .020 | .001 | .002 |
| Residual | 5176.03 (2263) | .991 | .022 | .001 | .001 | 7400.70 (4478) | .991 | .022 | .002 | .002 |
| **2 Factors (Model 3)** |  |  |  |  |  |  |  |  |  |  |
| Configural | 2965.28 (2043) | .997 | .013 |  |  | 4187.50 (4086) | .999 | .014 |  |  |
| Scalar | 3038.56 (2075) | .997 | .013 | .000 | .000 | 4478.11 (4164) | .999 | .016 | .000 | .002 |
| Residual | 3314.09 (2139) | .997 | .014 | .000 | .001 | 5969.69 (4292) | .995 | .017 | .004 | .001 |

**Table 1s**. Measurement invariance based on complete cases (*N* = 2684)

*Note*. CFI = Comparative Fit-Index; RMSEA = Root Mean Square Error of Approximation; *df* = degrees of freedom

*Here, we allowed for the covariances of errors between the reversely coded items.

ΔCFI ≥ .010 and ΔRMSEA ≥ .007 indicate substantial deterioration in model fit (Neufeld et al, under review). Models are compared with the prior model consisting of one less level of constraints.

**Table s2.** Correlations between sum scores of the total scores and the two-factors affective depression and somatic symptoms

|  | **1T** | **2T** | **3T** | **4T** | **5T** | **6T** | **7T** | **8T** | **9T** | **1A** | **2A** | **3A** | **4A** | **5A** | **6A** | **7A** | **8A** | **9A** | **1S** | **2S** | **3S** | **4S** | **5S** | **6S** | **7S** | **8S** | **9S** |
| --- | --- | --- | --- | --- | --- | --- | --- | --- | --- | --- | --- | --- | --- | --- | --- | --- | --- | --- | --- | --- | --- | --- | --- | --- | --- | --- | --- |
| **1T** | **-** |  |  |  |  |  |  |  |  |  |  |  |  |  |  |  |  |  |  |  |  |  |  |  |  |  |  |
| **2T** | .53 | **-** |  |  |  |  |  |  |  |  |  |  |  |  |  |  |  |  |  |  |  |  |  |  |  |  |  |
| **3T** | .52 | .55 | - |  |  |  |  |  |  |  |  |  |  |  |  |  |  |  |  |  |  |  |  |  |  |  |  |
| **4T** | .46 | .50 | .55 | **-** |  |  |  |  |  |  |  |  |  |  |  |  |  |  |  |  |  |  |  |  |  |  |  |
| **5T** | .45 | .49 | .50 | .55 | - |  |  |  |  |  |  |  |  |  |  |  |  |  |  |  |  |  |  |  |  |  |  |
| **6T** | .42 | .43 | .47 | .50 | .56 | - |  |  |  |  |  |  |  |  |  |  |  |  |  |  |  |  |  |  |  |  |  |
| **7T** | .41 | .42 | .44 | .49 | .50 | .58 | - |  |  |  |  |  |  |  |  |  |  |  |  |  |  |  |  |  |  |  |  |
| **8T** | .40 | .40 | .43 | .44 | .46 | .53 | .57 | - |  |  |  |  |  |  |  |  |  |  |  |  |  |  |  |  |  |  |  |
| **9T** | .36 | .37 | .39 | .45 | .46 | .48 | .51 | .57 | - |  |  |  |  |  |  |  |  |  |  |  |  |  |  |  |  |  |  |
| **1A** | .90 | .47 | .45 | .38 | .37 | .34 | .35 | .34 | .31 | - |  |  |  |  |  |  |  |  |  |  |  |  |  |  |  |  |  |
| **2A** | .48 | .90 | .49 | .44 | .41 | .36 | .37 | .34 | .31 | .48 | - |  |  |  |  |  |  |  |  |  |  |  |  |  |  |  |  |
| **3A** | .46 | .48 | .90 | .48 | .43 | .41 | .38 | .37 | .33 | .44 | .48 | - |  |  |  |  |  |  |  |  |  |  |  |  |  |  |  |
| **4A** | .40 | .44 | .49 | .91 | .48 | .43 | .42 | .36 | .36 | .37 | .43 | .48 | - |  |  |  |  |  |  |  |  |  |  |  |  |  |  |
| **5A** | .40 | .43 | .45 | .49 | .89 | .49 | .45 | .39 | .40 | .38 | .41 | .43 | .49 | - |  |  |  |  |  |  |  |  |  |  |  |  |  |
| **6A** | .36 | .37 | .40 | .43 | .50 | .90 | .49 | .44 | .41 | .34 | .35 | .39 | .42 | .49 | - |  |  |  |  |  |  |  |  |  |  |  |  |
| **7A** | .35 | .36 | .37 | .41 | .43 | .51 | .88 | .47 | .42 | .33 | .36 | .36 | .41 | .44 | .49 | - |  |  |  |  |  |  |  |  |  |  |  |
| **8A** | .35 | .36 | .37 | .37 | .40 | .47 | .49 | .88 | .49 | .33 | .33 | .35 | .35 | .39 | .45 | .47 | - |  |  |  |  |  |  |  |  |  |  |
| **9A** | .32 | .33 | .34 | .37 | .39 | .41 | .45 | .50 | .88 | .31 | .31 | .39 | .36 | .39 | .42 | .45 | .51 | - |  |  |  |  |  |  |  |  |  |
| **1S** | .84 | .47 | .46 | .41 | .42 | .39 | .37 | .36 | .33 | .53 | .36 | .35 | .33 | .32 | .29 | .27 | .27 | .25 | - |  |  |  |  |  |  |  |  |
| **2S** | .45 | .84 | .46 | .44 | .44 | .39 | .37 | .38 | .34 | .33 | .52 | .33 | .32 | .32 | .29 | .25 | .28 | .25 | .48 | - |  |  |  |  |  |  |  |
| **3S** | .45 | .48 | .84 | .49 | .45 | .42 | .39 | .39 | .35 | .33 | .36 | .53 | .36 | .33 | .31 | .27 | .28 | .25 | .48 | .49 | - |  |  |  |  |  |  |
| **4S** | .40 | .43 | .47 | .84 | .48 | .44 | .43 | .41 | .38 | .28 | .31 | .33 | .52 | .35 | .31 | .30 | .29 | .28 | .43 | .46 | .50 | - |  |  |  |  |  |
| **5S** | .38 | .42 | .44 | .48 | .83 | .48 | .43 | .41 | .40 | .26 | .31 | .32 | .34 | .53 | .36 | .30 | .34 | .29 | .42 | .45 | .47 | .51 | - |  |  |  |  |
| **6S** | .37 | .38 | .41 | .44 | .48 | .85 | .51 | .48 | .42 | .26 | .28 | .31 | .32 | .34 | .51 | .37 | .35 | .31 | .41 | .41 | .43 | .47 | .50 | - |  |  |  |
| **7S** | .36 | .36 | .38 | .43 | .43 | .49 | .83 | .50 | .45 | .26 | .26 | .28 | .30 | .32 | .34 | .48 | .36 | .32 | .38 | .39 | .40 | .46 | .44 | .52 | - |  |  |
| **8S** | .34 | .34 | .37 | .39 | .39 | .44 | .48 | .84 | .47 | .24 | .23 | .27 | .26 | .26 | .29 | .32 | .48 | .33 | .36 | .37 | .39 | .43 | .43 | .49 | .52 | - |  |
| **9S** | .29 | .30 | .33 | .35 | .39 | .39 | .41 | .47 | .83 | .22 | .21 | .24 | .25 | .28 | .27 | .36 | .32 | .47 | .31 | .34 | .35 | .39 | .42 | .42 | .46 | .49 | - |

**Note.** T = total score, A = affective score, S = somatic score, all *p* ≤ .001**.**
